# Treatment results of diabetic macular edema with different choroidal thickness with intravitreal anti vascular endothelial growth factor

**DOI:** 10.1186/s12886-022-02721-3

**Published:** 2022-12-22

**Authors:** Fatma Savur, Havva Kaldırım, Kürşat Atalay, Tülin Öğreden, Şerife Çiloğlu Hayat

**Affiliations:** 1 Ophthalmology Department, Istanbul Health Sciences University, Bagcilar Training and Research Hospital, Istanbul, Turkey; 2 Ophthalmology Department, Istanbul Health Sciences University, Basaksehir Cam and Sakura City Hospital, Basaksehir, P.O. Box 34200, Istanbul, Turkey

**Keywords:** Anti-vascular endothelial growth factor, Choroidal thickness, Diabetic macular edema, Macular thickness

## Abstract

**Purpose:**

To compare the results of intravitreal anti-vascular endothelial growth factor (anti-VEGF) therapy in patients with diabetic macular edema (DME) with different choroidal thicknesses.

**Methods:**

The files of patients diagnosed with DME and treated with intravitreal anti-VEGF were reviewed retrospectively. The best-corrected visual acuity (BCVA), choroidal thickness (CT), and macular thickness (MT) measurements were recorded before and after treatment. All patients included in the study were divided into 3 groups according to the initial subfoveal choroidal thickness (SFCT). Group 1 included 35 patients with SFCT ≤ 220, group 2 included 27 patients with SFCT > 220 ≤ 270, and group 3 included 30 patients with SFCT > 270. The total number of anti-VEGF administered during the follow-up at the last examination, baseline and post-treatment CT, MT, and BCVA measurements were statistically compared in all 3 groups.

**Results:**

The mean age of the patients was 61.9 ± 10.2 in group 1, 58.7 ± 8.7 in group 2, and 57.0 ± 6.5 in group 3. The mean anti-VEGF count in group 1 was significantly lower than group 2 and group 3 (*p* = 0.004, *p* = 0.006). In Group 1, BCVA improved significantly after treatment compared to baseline (*p* = 0.001). In Groups 2 and 3, BCVA did not change significantly after treatment compared to baseline (*p* = 0.320, *p* = 0.104). After treatment, central macular thickness decreased significantly in group 1 compared to baseline, while central macular thickness did not show a significant change from baseline in group 2 and group 3 after treatment (*p* = 0.003, *p* = 0.059, *p* = 0.590).

**Conclusion:**

In our study, we observed that the treatment needs of our DME patients with different choroidal thicknesses were different. In patients with DME, the initial choroidal thickness may help determine the need for follow-up and treatment.

## Introduction

Diabetic macular edema (DME) is the most important and most common cause of vision loss in people with diabetes mellitus [1]. In Europe, it is estimated that people with diabetes affected by any diabetes-related eye disease will increase from 6.4 million today to 8.6 million in 2050, 30% of whom will require close monitoring and/or treatment [2]. Uncontrolled hyperglycemia causes a decrease in inner retinal oxygen pressure and venous dilatation, an increase in vascular endothelial growth factor (VEGF) concentration in the retina, leukocyte stasis, and capillary permeability, and DME. Ocular treatments for DME include intravitreal injections of drugs (anti-VEGF and/or corticosteroids), focal laser photocoagulation, and vitrectomy, but a significant proportion of eyes do not respond fully to the treatments administered. A change or combination of existing treatment methods can be applied in this case. While focal/grid laser photocoagulation and intravitreal corticosteroid injections have been used in the treatment of DME in the past, intravitreal anti-VEGF drugs are considered first-line therapy today. Given the chronic course of diabetes and the short-term effects of intravitreal anti-VEGFs, frequent patient visits and repeated anti-VEGF injections are necessary to maintain success in DME treatment. Although intravitreal anti-VEGF therapy can improve vision in DME, the high cost and time burden of frequent visits and injections prompt us to seek different treatment options and approaches. It is also believed that frequent monitoring and/or treatment burdens are the main causes of inadequate treatment. Given the increasing incidence of diabetes worldwide, a treatment regimen that maximizes vision potential, improves compliance, and reduces costs would be ideal.

In our study, we evaluated the relationship between subfoveal choroidal thickness (SFCT) and anti-VEGF therapy in patients with DME and investigated whether subfoveal choroidal thickness could help determine the treatment modality of patients with DME.

## Material and methods

In this study, patients diagnosed with DME and treated with intravitreal anti-VEGF in the eye clinic retina at the Department of Health Sciences, Bağcılar University Training and Research Hospital were included. Written consent forms were obtained from the patients participating in the study. The study was conducted following the principles of the Declaration of Helsinki.

We recorded the demographic and clinical data of all patients, including age, gender, hemoglobin A1c (HbA1c), number and type of anti-VEGF treatments administered, and baseline and final (last follow-up) best-corrected visual acuity (BCVA) in logMAR. Dilated fundus examination and fundus fluorescein angiography (FFA) findings of all patients included in the study were evaluated. Heidelberg Spectralis (Spectralis®, Heidelberg Engineering, Heidelberg, Germany, Software version 5.0), advanced depth imaging-optical coherence tomography (EDI-OCT) images were taken according to the method described previously, and horizontal sections from the foveas were examined [3]. DME was defined as a diffuse thickening or cystic change of 250 µm or more in the fovea (500 µm region) on OCT. To measure subfoveal choroidal thickness, the distance from the inner sclera to the hyperreflective outer edge of the retinal pigment epithelium was assessed manually on EDI-OCT images using the Image J tool (National Institute of Health, Bethesda, MD, USA. Choroidal thickness (CT) and macular thickness (MT) were measured nasally and temporally in the subfoveal area 1 mm from the center of the fovea. Each measurement before and after treatment was made by two experienced, blinded investigators. The average of the two measurements obtained in µm was used for the final statistical analysis.

All patients included in the study were untreated (naïve) patients with type 2 diabetic macular edema. Patients diagnosed with DME were treated with a 3-month loading dose of intravitreal anti-VEGF (ranibizumab or aflibercept) followed by pro-re-nata (PRN). The patients included in the study were divided into three groups according to their baseline subfoveal choroidal thickness. Group 1 included 35 patients with SFCT ≤ 220, Group 2 included 27 patients with SFCT > 220 ≤ 270, and Group 3 included 30 patients with SFCT > 270. The total amounts of anti-VEGF administered during the follow-up at the last examination and initial and final CT, MT, and BCVA measurements were statistically compared in all three groups.

Patients with proliferative diabetic retinopathy (PDR), macular scarring, FFA-detected macular ischemia, previous laser therapy, previous intravitreal anti-VEGF or steroid injection, high refractive error (< -5.00 D, >  + 5.00 D), glaucoma, uveitis, vitreomacular interface abnormalities, kidney disease, and other causes of retinopathy, such as hypertension, significant cataracts, and any history of intraocular surgery within the past year, along with epiretinal patients with membranes, were excluded from the study.

### Statistical methods

The mean, standard deviation, median, minimum, maximum value frequency, and percentage were used for the descriptive statistics. The distribution of variables was checked using the Kolmogorov–Smirnov test. ANOVA, Kruskal–Wallis, and Mann–Whitney U tests were used for the comparison of quantitative data. A paired samples t-test and Wilcoxon test were used for the repeated measurement analysis. The chi-square test was used for the comparison of qualitative data. Multiple comparison tests were used in our study. SPSS 27.0 was used for statistical analyses.

## Results

The mean age of the patients was 61.9 ± 10.2 in Group 1, 58.7 ± 8.7 in Group 2, and 57.0 ± 6.5 in Group 3. The mean age of the patients in Group 1 was significantly higher than that in Group 3 (*p* = 0.002). The demographic data of the patients are summarized in Table 1. The mean age of the patients in Group 2 did not differ significantly from that in Groups 1 and 3 (*p* = 0.087, *p* = 0.270, respectively). The mean follow-up time did not differ significantly between the groups (*p* = 0.679). The mean anti-VEGF count in Group 1 was significantly lower than that in Groups 2 and 3 (*p* = 0.004, *p* = 0.006). There was no significant difference between the mean amount of anti-VEGF in Groups 2 and 3 (*p* = 0.711). Best-corrected visual acuity did not differ significantly between the groups at baseline and after treatment (*p* = 0.056, *p* = 0.748) (Table 2). In Group 1, BCVA improved significantly after treatment compared to the baseline (*p* = 0.001). In Groups 2 and 3, BCVA did not change significantly after treatment compared to the baseline (*p* = 0.320, *p* = 0.104). The change in BCVA after treatment in Group 1 was significantly higher than that in Group 2 (*p* = 0.008). The change in BCVA after treatment in Group 3 did not differ significantly from that in Groups 1 and 2 (*p* = 0.164, *p* = 0.085).

**Table 1:** Summary of the demographic data

		GROUP 1				GROUP 2				GROUP 3					
		Mean ± s.d /n-%			Med	Mean ± s.d /n-%			Med	Mean ± s.d /n-%			Med		
Age		61.9	±	10.2	65.0	58.7	±	8.7	58.0	57.0	±	6.5	57.0	***0.009***	^K^
Gender	Female	19		54.3%		13		48.1%		13		41.9%		0.605	^X2^
	Male	16		45.7%		14		51.9%		18		58.1%			
Number of Anti-VEGF		5.4	±	1.8	5.0	7.1	±	2.4	7.0	7.0	±	2.7	7.0	***0.004***	^K^
HbA1c		8.0	±	2.1	8.2	8.2	±	2.2	7.7	8.3	±	2.0	8.2	0.872	^K^
Anti-VEGF Name	Aflibercept	19		54.3%		10		37.0%		15		48.4%		0.398	^X2^
	Ranibizumab	16		45.7%		17		63.0%		16		51.6%			
Follow-Up Month		25.1	±	1.7	25.0	24.8	±	1.5	25.0	25.2	±	1.4	25.0	0.679	^K^

GROUP 1: SFCT ≤ 220

GROUP 2: 220 < SFCT ≤ 270

GROUP 3: SFCT > 270

^K^Kruskal–wallis (Mann–whitney u test) / ^X2^Chi-square test

**Table 2:** Intergroup comparison of baseline and final best-corrected visual acuity and nasal, subfoveal, and temporal macular and choroidal thickness

	GROUP 1				GROUP 2				GROUP 3					
	Mean ± s.d /n-%			Med	Mean ± s.d /n-%			Med	Mean ± s.d /n-%			Med		
***Best Corrected Visual Acuity***														
Baseline	0.64	±	0.51	0.50	0.37	±	0.37	0.30	0.47	±	0.37	0.40	0.056	^K^
Final	0.34	±	0.23	0.30	0.43	±	0.36	0.30	0.40	±	0.38	0.30	0.748	^K^
Baseline/Final Difference	-0.30	±	0.44	-0.08	0.06	±	0.33	0.00	-0.08	±	0.33	0.00	***0.008***	^K^
Intra Group p Value	***0.001***			^W^	0.320			^W^	0.104			^W^		
***Nasal Choroidal Thickness***														
Baseline	174.5	±	30.1	178.0	234.2	±	29.5	235.0	274.8	±	32.7	271.0	***0.000***	^A^
Final	179.3	±	43.7	178.0	221.0	±	27.2	225.0	261.3	±	32.2	259.0	***0.000***	^A^
Baseline/Final Difference	4.8	±	31.1	6.0	-13.1	±	30.9	-12.0	-13.5	±	43.2	-15.0	0.063	^A^
Intra Group p Value	0.367			^P^	***0.036***			^P^	***0.042***			^P^		
***Subfoveal Choroidal Thickness***														
Baseline	190.2	±	30.4	198.0	247.2	±	14.2	249.0	296.2	±	25.1	291.0	***0.000***	^A^
Final	192.0	±	39.6	195.0	230.7	±	42.0	238.0	281.8	±	33.3	283.0	***0.000***	^A^
Baseline/Final Difference	1.8	±	28.5	0.0	-16.5	±	40.8	-14.0	-14.4	±	34.7	-17.0	0.070	^A^
Intra Group p Value	0.707			^P^	***0.046***			^P^	***0.028***			^P^		
***Temporal Choroidal Thickness***														
Baseline	180.8	±	29.1	180.0	230.1	±	30.7	234.0	270.0	±	28.0	265.0	***0.000***	^A^
Final	184.1	±	40.0	196.0	220.2	±	29.4	215.0	259.0	±	34.7	255.0	***0.000***	^A^
Baseline/Final Difference	3.3	±	30.3	-1.0	-9.9	±	35.1	-13.0	-11.0	±	34.5	-15.0	0.156	^A^
Intra Group p Value	0.522			^P^	0.156			^P^	0.086			^P^		
***Nasal Macular Thickness***														
Baseline	391.3	±	79.3	351.0	376.9	±	59.9	371.0	383.3	±	59.3	369.0	0.875	^K^
Final	340.9	±	48.3	334.0	359.0	±	70.5	343.0	380.9	±	64.4	356.0	0.051	^K^
Baseline/Final Difference	-50.5	±	75.0	-39.0	-17.9	±	57.8	-13.0	-2.4	±	69.7	-2.0	0.052	^K^
Intra Group p Value	***0.001***			^W^	***0.049***			^W^	0.814			^W^		
***Central Macular Thickness***														
Baseline	359.4	±	135.4	310.0	317.7	±	71.2	304.0	349.6	±	128.9	311.0	0.808	^K^
Final	279.9	±	78.0	265.0	294.6	±	83.9	275.0	343.0	±	117.6	309.0	0.052	^K^
Baseline/Final Difference	-79.5	±	144.5	-42.0	-23.1	±	57.8	-14.0	-6.7	±	122.8	-4.0	0.130	^K^
Intra Group p Value	***0.003***			^W^	0.059			^W^	0.590			^W^		
***Temporal Macular Thickness***														
Baseline	377.8	±	82.1	346.0	344.4	±	38.6	352.0	363.7	±	57.4	348.0	0.674	^K^
Final	334.4	±	58.8	326.0	334.8	±	47.2	328.0	383.8	±	68.6	369.0	***0.002***	^K^
Baseline/Final Difference	-43.5	±	78.7	-24.0	-9.6	±	47.0	-4.0	20.1	±	69.0	6.0	***0.002***	^K^
Intra Group p Value	***0.000***			^W^	***0.000***			^W^	***0.000***			^W^		

^A^ANOVA / ^K^Kruskal–wallis (Mann–whitney u test) / ^w^Wilcoxon test / ^P^Paired Samples t test

Choroidal thickness in the nasal, subfoveal, and temporal quadrants was significantly higher in Group 3 compared to Groups 1 and 2 at the baseline and after treatment (*p* < 0.05). The choroidal thickness in the nasal, subfoveal, and temporal quadrants was significantly higher in Group 2 than in Group 1 at the baseline and after treatment (*p* < 0.05). After treatment, nasal and subfoveal CT did not significantly change from the baseline in Group 1 (*p* = 0.367, *p* = 0.707), while they decreased significantly in Groups 2 and 3 (*p* = 0.036, *p* = 0.046, *p* = 0.042, *p* = 0.028). After treatment, temporal CT did not change significantly from the baseline in Groups 1, 2, or 3 (*p* = 0.522, *p* = 0.156, *p* = 0.086). The changes in nasal, temporal, and subfoveal choroidal thickness after treatment did not differ significantly between the groups (*p* = 0.156).

Nasal and central MT did not differ significantly between the groups at the baseline and after treatment (*p* = 0.875, *p* = 0.808, *p* = 0.051, *p* = 0.052). Temporal MT did not differ significantly between the groups at the baseline (*p* = 0.674, *p* = 0.002). After treatment, the temporal MT was significantly higher in Group 3 than in Groups 1 and 2 (*p* = 0.002). Temporal macular thickness did not differ significantly in Groups 1 and 2 after treatment (*p* = 0.972). While there was a significant decrease from the baseline in nasal macular thickness after treatment in Groups 1 and 2, no significant change was observed in Group 33 (*p* = 0.001, *p* = 0.049, *p* = 0.814). After treatment, the central macular thickness decreased significantly in Group 1 compared to the baseline, while central macular thickness did not show a significant change from the baseline in Groups 2 and 3 after treatment (*p* = 0.003, *p* = 0.059, *p* = 0.590). After treatment, temporal MT decreased significantly in Groups 1 and 2 compared to the baseline, while in Group 3, temporal macular thickness increased significantly after treatment (*p* < 0.05). After treatment, the nasal and central MT changes did not differ significantly between the groups (*p* = 0.052, *p* = 0.130). The temporal MT change after treatment was significantly higher in Group 3 than in Group 1 (*p* = 0.001). The change in temporal macular thickness after treatment did not differ significantly in Group 2 compared to Groups 1 and 3(*p* = 0.060, *p* = 0.098).

## Discussion

The basis of the pathogenesis of DME is increased vascular permeability in the retinal vessels. As is known, the nutrition and oxygenation of the photoreceptor cells in the outer layer of the retina and the retinal pigment epithelium are provided by choroidal blood flow. Therefore, the choroid plays an important role in the pathology of many retinal diseases, including diabetic retinopathy (DR). In a previous study, the choroids of diabetic patients were examined using light and electron microscopy, and it was reported that choroidal vascular changes caused by diabetes were similar to vascular changes that occur in other tissues of the body and other layers of the eye [4]. These vascular changes in the choroids of patients with diabetic retinopathy significantly reduce subfoveal choroidal blood flow. Nagaoka et al. observed that choroidal blood flow in the fovea regions of patients with type 2 diabetes was significantly reduced compared to the control group, especially in DME [5].

Ranibizumab, an anti-VEGF, has been widely used in the treatment of DME since receiving FDA approval for intravitreal administration in 2011. Many studies have been conducted to evaluate the efficacy and different combinations of intravitreal anti-VEGFs to achieve optimal success, as they are currently considered the first treatment option in DME. Studies have shown that intravitreal administration of ranibizumab alone is superior to macular laser photocoagulation in DME [6–8]. In the RISE/RIDE Phase 3 study, in which different doses of intravitreal ranibizumab and sham injection were administered monthly, ranibizumab was shown to be effective in improving macular edema and BCVA. A delay in the initiation of ranibizumab therapy has been reported to result in a limited improvement in BCVA at three years [9].

Bevacizumab is frequently used off-label in the treatment of DME due to its cost-effectiveness, and its efficacy has been demonstrated in clinical studies [10, 11]. In a prospective clinical study comparing intravitreal bevacizumab therapy with laser therapy alone in DME, the mean BCVA was significantly better in the bevacizumab-administered group compared to laser therapy alone [10].

After all these studies, aflibercept, another anti-VEGF agent developed to reduce the frequency of intravitreal injections, was approved by the FDA for the treatment of DME in 2014 [12]. In the DRCR.net Protocol T study comparing intravitreal administration of aflibercept, bevacizumab, and ranibizumab, all three anti-VEGFs improved visual acuity in eyes with DME. While there was no statistically significant difference in efficacy between the three anti-VEGFs in patients with better initial visual acuity, aflibercept was statistically significantly more effective than bevacizumab and ranibizumab in patients with poor baseline visual acuity. The mean number of intravitreal injections administered was 9 in the aflibercept group, 10 in the bevacizumab group, and 10 in the ranibizumab group [13].

Currently, the standard treatment option for DME in developed countries is recurrent anti-VEGF intravitreal injection monotherapy. Repeated intravitreal anti-VEGF injections cause frequent hospital visits, heavy treatment costs, and losses in workforce for both patients and their attendants. This situation reduces the compliance of patients with the treatment and causes them to interrupt the treatment. The VISTA and VIVID studies compared the results of the administration of intravitreal aflibercept at a dose of 2 mg at four-week (IAI 2q4) and eight-week intervals (IAI 2q8) (after five initial monthly injections). The IAI 2q4 and IAI 2q8 regimens were shown to have similar efficacy. It was concluded that the IAI 2q8 regimen may have the potential to reduce the treatment burden in most patients with DME [14]. In a study comparing different administration regimens of intravitreal ranibizumab therapy in DME, patients with the treat-and-extend (TE) dose regimen required 40% fewer treatment visits compared to the pro re nata regimen. Of the patients, 70% had an interval of visits lasting more than two months. An important limitation of this study is that it did not include a patient group undergoing a monthly treatment regimen [15].

In all these studies, the aim is to obtain optimal visual acuity with different anti-VEGF agents used in DME and their different treatment regimens, with few hospital visits and injections. Thus, the goal is to prevent losses in the workforce by increasing patient compliance and quality of life. Unfortunately, unlike these randomized controlled trials (RCTs), the results achieved with DME treatment in real-world conditions are not very good. In real-world data, patients have been reported to be less compliant and to have lower visual acuity gains due to fewer intravitreal injections [16–21]. In a retrospective study by Kiss et al., the mean number of intravitreal bevacizumab injections administered to 2,733 newly diagnosed DME patients in 2008, 2009, and 2010 was 2.2, 2.5, and 3.6, respectively [22]. Aken et al. reported 5.1 ± 3.0 ranibizumab injections at an average of 9.9 ± 3.5 visits per year in patients with naive DME [23]. Habib et al. reported the average number of injections as 1.32 ± 0.65 in a study in which they examined the factors affecting injection compliance. It has also been concluded that financial and psychological burdens may be the main controllable factors in patient compliance with anti-VEGF therapy in DME [24]. In the multicenter POLARIS study examining ranibizumab injections in DME, the mean number of injections was 4.5 ± 2.3 in all patients, and the mean injection rate was 2.0 ± 1.2 in Russia and 6.7 ± 2.3 in the UK [25].

This low level of patient compliance and treatment success in real life and the increase in treatment costs motivate the search for different options and approaches for the treatment of DME. In light of this information, we examined the choroidal thicknesses of patients who had previously been treated with anti-VEGF for DME treatment. We wanted to evaluate whether choroidal thickness has prognostic value in the anti-VEGF treatment of DME. As we mentioned earlier, diabetic retinopathy causes choroidal changes called diabetic choroidopathy as well as changing the retina [4]. The introduction of advanced depth imaging-optical coherence tomography, which has been developed in recent years and enables us to obtain high-quality and -resolution macular cross-section images, has helped us monitor the changes in the choroid [4]. Thanks to the EDI module, intensive studies on the choroid have helped us understand its role in the pathogenesis of many chorioretinal diseases. Research on this subject continues intensively today. Mathis et al. reported that CT increased during the recurrence of DME in patients who had previously received intravitreal anti-VEGF or dexamethasone therapy for DME. They suggested that this increase in CT could be a new indicator for monitoring patients with DME. They suggested that vasodilation of the choroidal vessels due to increased VEGF causes choroidal thickening at recurrence, increasing CT [26]. Based on these data, we divided the patients who received intravitreal anti-VEGF therapy for DME treatment in our clinic into three groups according to their initial choroidal thickness.

The mean number of injections with thin choroidal thickness in Group 1 was also significantly less than that in Groups 2 and 3(mean 5.4, 7.1, 7.0 respectively). There was no significant difference between the mean number of anti-VEGF in Groups 2 and 3 (*p* > 0.05). In Group 1, which had thin choroidal thickness, we observed a reduction in macular thickness with less anti-VEGF administration without a significant reduction in choroidal thickness after treatment. The BCVA did not differ significantly between the groups at baseline and after treatment (*p* > 0.05). In Group 1, BCVA improved significantly after treatment compared to the baseline (*p* < 0.05). In Groups 2 and 3, BCVA did not change significantly after treatment compared to the baseline (*p* > 0.05). Nasal, central, and temporal MT was similar between the groups at the beginning, while temporal MT was significantly higher in Group 3 after treatment than in Groups 1 and 2 (*p* > 0.05, *p* < 0.05). Although the mean number of injections was higher in Group 3, the decrease in MT was significantly lower than in the other groups. This suggests that increased CT may be an indicator of the need for anti-VEGF injections and resistance to therapy.

## Conclusion

In our study, better visual gain was achieved with fewer intravitreal anti-VEGF injections in DME patients with thin CT. For DME patients with thinner baseline CT, fewer visits and treatments can help reduce patient compliance and treatment costs. The conclusion that subfoveal CT may be a prognostic factor in the application of anti-VEGF in the treatment of DME needs to be supported by larger prospective controlled studies.

## Data Availability

The datasets used and/or analysed during the current study are available from the corresponding author on reasonable request.
